# Management of Sport Injuries with Korean Medicine: A Survey of Korean National Volleyball Team

**DOI:** 10.1155/2016/8639492

**Published:** 2016-08-29

**Authors:** Changsop Yang, Eunyoung Lee, Eui-Hyoung Hwang, Ojin Kwon, Jun-Hwan Lee

**Affiliations:** ^1^Clinical Research Division, Korea Institute of Oriental Medicine, Daejeon 34054, Republic of Korea; ^2^Mibyeong Research Center, Korea Institute of Oriental Medicine, Daejeon 34054, Republic of Korea; ^3^Third Division of Clinical Medicine, School of Korean Medicine, Pusan National University, Pusan 50612, Republic of Korea; ^4^Korean Medicine Life Science, University of Science & Technology (UST), Campus of Korea Institute of Oriental Medicine, Daejeon 34054, Republic of Korea

## Abstract

The purpose of this study was to report the current state of Korean medicine (KM) treatment on sports injury by implementing survey with volleyball team medical doctors participating in 2013-2014 season. Six KM doctors completed a questionnaire that includes injury parameters: type, location, situation, and pain scores. We collected 166 injury cases from 94 Korean male and female national volleyball players. Knee (25.9%), low back (13.3%), elbow, and ankle (8.4%) injuries were most common. Joint (41.6%) and muscle (30.7%) were major injured tissues. KM team medical doctors utilized acupuncture (40.4%), chuna manual therapy (16.0%), physical therapy (15.2%), taping (9.0%), and cupping (7.8%) to treat volleyball injuries. Any types of medications were used infrequently. Additional physical and exercise therapy were preferred after receiving acupuncture (both 46.9%). This study presented the preliminary injury profile of Korean elite volleyball players. Injury and treatment parameters could be useful to build advanced KM model in sport medicine.

## 1. Introduction

The Korean medicine (KM) shares the same therapeutic techniques with East Asian traditional medicine. Some techniques such as acupuncture and cupping are used worldwide now. It also has distinctive features such as sasang typology, saam acupuncture, chuna manual therapy, pharmacopuncture, and pattern identification [1]. Research of KM in the field of sport medicine has begun in the early 1990s to integrate KM with sports injury care and to establish a firm foundation with a genuine system and developmental plan. KM therapies including acupuncture, moxibustion, chuna manual therapy, pharmacopuncture, and herbal medicine were expected to be useful to treat sports injuries [2, 3]. However, there is a paucity of well-designed clinical KM research regarding sports injury [4, 5]. To fully integrate KM into sports injury treatment, it is imperative to link KM with established sports related studies.

Globally, volleyball is one of the most popular sports. The Federation Internationale de Volleyball includes 220 member federations and presides over 5 continental confederations. Volleyball is a powerful sport that involve repeated forceful arm actions and jumps that can lead to shoulder and knee injuries. Several epidemiological surveys have described preventive strategies based on the injury mechanisms [6–8]. However, there was no study focusing on volleyball injuries managed with KM. The present study was conducted to report the current state of KM treatment with regard to sports injury based on the results of a survey of national male and female volleyball team medical doctors participating in the 2013-2014 season.

## 2. Materials and Methods

### 2.1. Subjects

The subjects were six licensed KM doctors who participated as team medical doctors in 2013-2014 international volleyball season. They reported 166 injury profiles and treatment models using structured questionnaire. Written consents were obtained from all subjects. Personal information that can be used to identify an individual player was eliminated during data capturing.

### 2.2. Questionnaire

We developed a questionnaire to determine how KM volleyball team medical doctors treat sports injuries. It was developed in Korean language and queried the treatment backgrounds of the six doctors. Individual items were based on similar studies and modified to suit our subject matter [9].

The self-reported questionnaire included the following:

1. diagnosis given by team medical doctor and number of visits;
2. injury type and location and reason for visit;
3. injury duration, pain scores, injury mechanism, type of activity during injury, and game or training status;
4. previous treatment experience, reason for selecting particular treatment, and satisfaction;
5. need for imaging diagnosis;
6. treatment content and theoretical basis;
7. treatment place, condition, and required treatment time;
8. comanagement model.

*(I) Injury Questionnaire*

 Diagnosis condition/reason for this consultation:___
 (If there were more than two diagnoses, please fill out another questionnaire)
 Number of treatment sessions:___
 Injury type (please √ one box)
   □ Bone
   □ Joint (include ligament)
   □ Muscle
   □ Tendon
   □ Contusion
   □ Laceration
   □ Central/peripheral nervous system
   □ Other:___
 Location of injury (please √ one box)
   □ Head
   □ Neck
   □ Chest
   □ Rib
   □ Upper back
   □ Lower back
   □ Upper arm
   □ Elbow
   □ Forearm
   □ Wrist
   □ Palm
   □ Thumb
   □ Finger
   □ Thigh
   □ Knee
   □ Shin
   □ Ankle
   □ Heel
   □ Soles of the feet
   □ Toe
   □ Other:___
 Reason for presentation (please √ one box)
   □ New injury
   □ Aggravation or exacerbation of an existing injury that had not fully resolved
   □ Recurrence of a previous injury that had fully resolved
   □ Maintenance/preventive/asymptomatic care
   □ Illness
   □ Other:___
 How long has the player had this condition or pain for (please √ one box)
   □ 0–7 days
   □ 1–4 wks
   □ 1–3 mths
   □ 3–6 mths
   □ 6–12 mths
   □ 1-2 yrs
   □ 2+ yrs
 Please rate the degree of pain the player has for this condition (circle one number)
   0 (No pain)
   1
   2
   3
   4
   5
   6
   7
   8
   9
   10 (Worst pain)
 How did the injury occur? (please √ one box)
   □ Contact/physical collision with another player or object [Specify:___
   □ Non-contact/DID NOT involve physical contact [Specify:___
   □ Uncertain/the injury gradually developed [Specify:___
 Type of activity at the time of injury (please √ one box)
   □ Competition [Specify period of game □ First □ Second □ Third □ Forth □ Fifth]
   □ Training/Practice
   □ Other:___
 Did the player have to stop playing or training because of the injury? (please √ one box)
   □ Yes
   □ No
 If no, was the player restricted or limited from full participation? (please √ one box)
    □ Yes
   □ No
 What other practitioners has the player previously consulted for this condition? (please √)
   □ None
   □ Traditional Korean medical doctor
   □ Medical doctor
   □ Physical therapist
   □ Trainer/Exercise therapist
   □ Other:___
 What is the reason the player stated for selecting a specific treatment for this condition? (please √ one box)
   □ Nearby location/Convenience
   □ Experience treated by oneself/Satisfied or improved following previous treatment
   □ Suggestion of a coaching staff or surrounding people (family, team)
   □ Renown of treatment group
   □ Advertisement/Internet
   □ Other:___
   □ None
 If player was previously treated for this condition, how did they feel about the outcome? (please √ one box)
   □ Highly satisfied
   □ Satisfied
   □ Partially satisfied
   □ Not satisfied
   □ Not at all satisfied
   □ None

*(II) Diagnosis and Treatment Questionnaire*

 Was a referral for advanced imaging required? (please √ one box)
   □ Yes
   □ No
   [Specify: □ X-ray □ CT/MRI □ Ultrasound □ Other___
 What kind of treatment was provided? (please √)
   □ Acupuncture treatment (include pharmacopuncture therapy)
   □ Cupping therapy
   □ Moxibustion therapy
   □ Herbal medicine
   □ Physical therapy
   □ Chuna manual therapy
   □ Exercise therapy
   □ Taping
   □ Over-the-counter medication
   □ Prescription medication
   □ Consultation
   □ Other:___
 Which treatment was provided with theory? (please √ one box)
   □ Theory of Traditional Korean medicine
   [Specify: □ Meridian system demonstration □ Organ demonstration □ Eight demonstration □ Other___
   □ Western medicine
   □ Combined Traditional Korean medicine and western medicine/used both
   □ Other___
   □ None
 Where was the treatment administered? (please √ one box)
   □ Training location
   □ Match location
   □ Other:___
 When was treatment provided? (please √ one box)
   □ Pre training
   □ During training
   □ Post training
   □ Pre match
   □ During match
   □ Post match
   □ Other:___
 How many minutes did you spend treating this patient? (please √ one box)
   □ <5
   □ 6–10
   □ 11–15
   □ 16–20
   □ 20–30
   □ 31–45
   □ 45–60
   □ >60
 Was co-management with another health care provider required? (please √ one box)
   □ No
   □ Yes
   [Specify:___ □ Medical doctor □ Ambulance □ Hospital □ Physiotherapist □ Trainer/Exercise therapist □ Other___
 If applicable, was this provided at the event? (please √ one box)
   □ Yes
   □ No

### 2.3. Ethical Review

This study was approved by the institutional review board of the Korea Institute of Oriental Medicine.

## 3. Results

### 3.1. General Characteristic

Six KM team medical doctors reported 166 injury cases from Korean male and female national volleyball players. Total 94 players (age range of 17–33 years; male : female = 76 : 18) experienced one or more injuries during 2013-2014 season. Players visited KM team medical doctors with mean 2.18 sessions (maximum 10 sessions). Mean pain score at visit was 5.4 (0–10 numeric pain rating scale) and most severe pain was reported in new injuries of knee and head.

### 3.2. Injury Location

**Figure 1:**
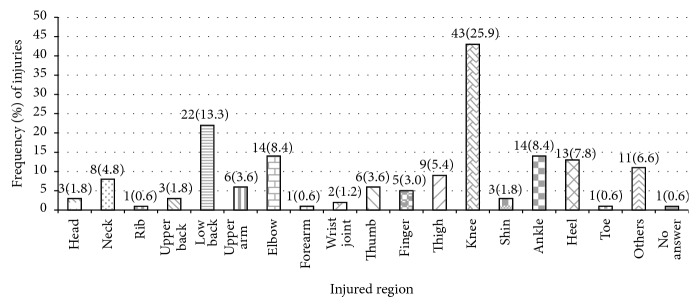
Location and frequency of injuries.

The most frequent injury site was the knee joint (43/166 cases, 25.9%) followed by the low back (22, 13.3%), elbow and ankle (each 14 cases, 8.4%), and heel (13 cases, 7.8%) (Figure 1). Gender differences were reported. Male players complained of knee (32 cases, 29.3%), low back (16 cases, 14.6%), and ankle (10 cases, 9.1%) injuries. Their knee injury was mainly recurrence or worsening of former damage (20 cases, 62.5%), while new damage was major cause (11 cases, 68.8%) in case of low back problem.

Female player complained of elbow (14 cases, 24.5%), knee (11 cases, 19.2%), and heel (8 cases, 14.0%) injuries. Female elbow and knee injuries were mainly caused by initial damage (12 cases, 85.7%, and 9 cases, 81.8%). Most frequent injured tissue was joint with 69 out of 166 cases (41.6%) followed by muscle with 51 cases (30.7%) and tendon with 14 cases (8.4%). Fracture was least frequent with 1 case (0.6%).

### 3.3. Distribution of Treatment Frequency by Injured Tissue

The most frequent treatment was acupuncture (151 cases, 40.4%) followed by chuna manual therapy (60 cases, 16.0%), physical therapy (57 cases, 15.2%), taping (34 cases, 9.0%), and cupping (29 cases, 7.8%). Korean over the counter (OTC) and prescribed medications were used infrequently. The injured tissue distribution revealed that acupuncture, chuna manual therapy, physical therapy, and taping were frequently used to treat joint injuries, while cupping was more used for treating muscle injury (Table 1).

### 3.4. Comanagement Therapy

No subjects required western medical assistant or emergency transfer after receiving treatment from KM team medical doctors. Two cases were reported to visit an external hospital after undergoing acupuncture, one case after physical therapy. Additional physical therapy and exercise were required after receiving acupuncture (23 cases each, 46.9%). No subject required additional physical therapy after receiving an initial session. After chuna manual therapy, 19 (54.3%) cases received physical therapy and 6 (17.1%) cases needed exercise based treatments from their trainers (Table 2).

## 4. Discussion

Beyond health benefits, the growing popularity of sports will result in increase of sport-induced injuries [10–13]. Advances in complementary and alternative medicine (CAM) techniques can have a major impact on modern sports medicine. In the field of professional and elite sports such as basketball, ice hockey, and wrestling, CAM treatment models are adopted to manage sport injuries [9, 14, 15].

The current KM system is being confronted by medical globalization and western-driven medical science development, making it difficult to maintain unique identity. Korean sports medicine is faced with a similar dilemma. There is lack of evidence reporting effectiveness of KM treating sport injury of elite sport player. Our survey of national male and female volleyball players would help clarify the role of Korean sports medicine.

This research was conducted to report the current state of KM treatment in sport injuries. It was carried out by using a questionnaire to assess the opinions of team medical doctors of national male and female volleyball players regarding various Korean sports medicine treatment methods and possible countermeasures. The questionnaire was designed in similar way as that employed in former sports injury studies [6, 9].

Volleyball involves repetitive movements, advanced technique, and excellent motor ability to spike, block, serve, receive, pass, and toss. Dynamic interplay involves various possible injuries. The most frequently injured location in this study was the knee joint (43/166 cases, 25.9%). Injuries of low back (22 cases, 13.3%), elbow and ankle (both 14 cases, 8.4%), and heel (13 cases, 7.8%) were reported more frequently than thumb (6 cases, 3.6%) and other fingers (5 cases, 3.0%) in this research. These results agree with former studies that described common injuries site including ankle, knee, and finger [6, 7, 16].

The most frequent treatment method was acupuncture (151 cases, 40.3%). As shown in Taiwanese wrestlers study, acupuncture has been used with sport injuries because it is rapid acting and easily applicable [15]. Acupuncture is effective for musculoskeletal disorders and there are positive reports on performance enhancement and postexercise recovery [14, 17–22].

Chuna manual therapy (60 cases, 16.0%) and physical therapy (57 cases, 15.2%) were also frequently used. Volleyball player repeatedly uses dominant shoulder and arm during serve or spike with extreme power. It affects muscle balance and range of motion of shoulder [23]. Chuna manual therapy is a KM therapy for pain control and repositioning of physical imbalance. Physical therapy is used less frequently because competitive provider such as western medical doctors and physiotherapist can use the same method. Taping therapy (34 cases) was the fourth most common treatment method; it was previously reported to have a beneficial effect on the knee joint [24]. However it was ranked fourth among the treatment methods, likely because it can be done by players themselves and does not require doctor's assistance. Cupping and moxibustion generally occupied low frequency and was applied in limited conditions. Medication (herbal, OTC, and prescription drugs) was infrequently used, possibly because players and coaches were cautious about unintended antidoping rule violation. KM team medical doctor preferred physical and exercise therapies as comanagement models coupled with acupuncture or chuna manual therapy.

This study is the first survey report that presents the preliminary injury and KM treatment profile of Korean elite volleyball players. Injury and treatment parameters could be useful to build advanced KM model in sport medicine. More large studies including wide range of volleyball player are necessary to indicate generalized trends of sport injury and KM therapeutic modalities.

## 5. Conclusion

Our study findings revealed that the knee and the low back were major lesions of volleyball injury among Korean elite players. Acupuncture, chuna manual therapy, and physical therapy are widely used by KM team medical doctors whereas moxibustion, medication, and exercise treatment are used infrequently. Acupuncture was often combined with physical and exercise therapies. This study might be useful to understand circumstances of KM in the field of volleyball injury. Further outcome study that shows best practice model for each sport injuries would be required. In addition, KM multimodal strategy to cooperate with other health professionals for treat and prevent sport injury will be also needed to achieve advancement of Korean sports medicine.

## Figures and Tables

**Table 1:** Treatment type for injured tissue.

Treatment	Category (total *n* = 166), *n* (%)			
	Joint	Muscle	Tendon	Others
Acupuncture	64 (38.1)	45 (41.3)	26 (44.8)	16 (41.0)
Cupping	12 (7.1)	16 (14.7)	0 (0.0)	1 (2.6)
Moxibustion	2 (1.2)	0 (0.0)	1 (1.7)	0 (0.0)
Herbal medicine	2 (1.2)	0 (0.0)	0 (0.0)	0 (0.0)
Physical therapy	26 (15.5)	22 (20.2)	6 (10.3)	3 (7.7)
Chuna manual therapy	32 (19.0)	9 (8.3)	10 (17.2)	9 (23.1)
Exercise treatment	7 (4.2)	4 (3.7)	1 (1.7)	1 (2.6)
Taping	16 (9.5)	7 (6.4)	10 (17.2)	1 (2.6)
Over-the-counter medication	3 (1.8)	2 (1.8)	1 (1.7)	4 (10.3)
Prescription medication	2 (1.2)	0 (0.0)	1 (1.7)	1 (2.6)
Consultation	0 (0.0)	0 (0.0)	0 (0.0)	1 (2.6)
Others	2 (1.2)	4 (3.7)	2 (3.4)	2 (5.1)

Total	168 (100.0)	109 (100.0)	58 (100.0)	39 (100.0)

Results are presented as number and percentage of cases.

**Table 2:** Comanagement after KM treatment.

Comanagement	Acupuncture	Physical therapy	Chuna manual therapy
Medical doctor	0 (0.0)	0 (0.0)	0 (0.0)
Ambulance	0 (0.0)	0 (0.0)	0 (0.0)
Hospital	2 (4.1)	1 (10.0)	0 (0.0)
Physiotherapist	23 (46.9)	0 (0.0)	19 (54.3)
Trainer	23 (46.9)	9 (90.0)	6 (17.1)
Not answered	1 (2.0)	0 (0.0)	0 (0.0)

Total	49 (100.0)	10 (100.0)	25 (100.0)

Results are presented as number and percentage of cases.
